# Supporting Nutritional Health of the UK Food Manufacturing Workforce: A Qualitative Study Exploring Insights From Employees

**DOI:** 10.1111/jhn.70310

**Published:** 2026-07-09

**Authors:** Georgia Rogerson, Bibi Rodgers Hunt, Louise Durrant, Hannah E. Theobald, Elena Philippou, Wendy L. Hall, Rachel Gibson

**Affiliations:** ^1^ Department of Nutritional Sciences, School of Life Course and Population Sciences King's College London London UK; ^2^ Marlow Foods Limited t/a Quorn Foods Stokesley North Yorkshire UK; ^3^ Department of Life Sciences, School of Life and Health Sciences University of Nicosia Nicosia Cyprus

**Keywords:** diet behaviour, food manufacturing, health promotion, occupational nutrition, qualitative interview, workplace health

## Abstract

**Introduction:**

Engagement of underserved groups, including low‐paid, shift workers, men, and ethnic minorities, is a priority for workplace health and wellbeing practitioners. The aims of the study were to explore experiences and perceptions around nutritional health and wellbeing in UK food manufacturing shift work employees and identify priorities to take forward for research, policy and practice.

**Methods:**

Online semi‐structured interviews with shift work food manufacturing workers were conducted between May and July 2025. Interview guides were developed using the Theoretical Domains Framework, aiming to explore factors influencing healthy nutrition choices when at work (the target behaviour) and to understand perceptions of worksite nutritional health and wellbeing provision. Data were analysed using a combined deductive framework and inductive thematic analysis approach; emergent themes were mapped to the COM‐B model and linked to potential Behaviour Change Techniques. Findings were externally validated with food and beverage industry stakeholders.

**Results:**

Interviews were conducted with 11 participants, 91% male, with a mean of 14 years of day and night shift work experience. Eight themes were identified including: (1) Having knowledge of healthy dietary choices for working on shift, (2) Physical consequences of dietary choices, (3) Intentions to make healthier dietary choices, (4) Influence of convenience on dietary choices, (5) Ability to access healthy dietary choices, (6) Social influence on dietary choices, (7) Emotional influence on dietary choices, (8) Ability to plan or monitor dietary intake.

**Conclusions:**

In addition to previously reported barriers around healthy food access while working atypical hours, UK food manufacturing staff face role‐specific challenges, including inadequate breaks, health and safety regulations and exposure to food production. Workplace policies to improve access to healthy nutrition and protected breaks may be prerequisites for individual‐level support for UK food manufacturing staff.

## Practitioner points

- Structural barriers exist for atypical shift workers that impede healthy dietary behaviours.
- Job role constraints require organisational‐level solutions to improve nutritional health for food manufacturing workers.
- Ensuring 24‐h access to healthy nutrition options is critical in the design of interventions in a shift‐work setting.

## 1 Introduction

The workplace offers a strategic setting for improving nutritional health, with evidence suggesting mutual benefits to worker health and business performance (e.g., reduced absenteeism, improved productivity [1]. In response to growing concerns around inclusivity, such as inequitable access or uptake in workplace health initiatives, the Nutrition Society's Workplace Diet and Health Special Interest Group convened a Round Table Meeting in 2023, identifying a key research priority: improving engagement among underserved employee groups, including low‐paid workers, men, shift workers, and individuals from ethnic minority backgrounds [2]. This also aligns with the Men's Health Strategy for England, which highlights less engagement with health promotion among men [3]. The UK's food and beverage manufacturing sector employs around 400,000 people [4], many in low‐paid, insecure roles with atypical hours. These working conditions are linked to poorer health outcomes and contribute to health inequalities [5]. There is limited evidence on how employers support nutritional health, particularly among low‐paid and shift‐based workers. Understanding current practices, barriers, and facilitators to delivery is essential to inform the design of inclusive and effective workplace nutrition programmes.

The overall aim of the ‘Ensuring the nutritional health of UK food manufacturing employees: Building evidence‐driven behaviour change interventions amongst a hard‐to‐reach occupational groups’ project is to understand current provision of workplace nutritional health interventions in the UK food and beverage manufacturing sector and how well such interventions meet the needs of shift workers.

A prior scoping review of 19 peer‑reviewed studies and 12 case studies reporting nutrition and wellbeing interventions conducted in food manufacturers identified 6 studies on nutrition and wellbeing interventions in UK food manufacturing [6]. The review found that interventions most commonly targeted behavioural antecedents, environmental restructuring and knowledge provision, with fewer than half reporting positive dietary change outcomes. Barriers to implementation occurred primarily at employee (e.g., engagement, language, resistance to change) and organisational levels (e.g., time, staffing and resources). Survey findings from UK practitioners working in UK food manufacturers aligned with the literature, highlighting antecedent‑focused approaches and identifying budget availability and senior management support as key enablers of implementation. Following on from this, the aims of the present study were to (i) explore experiences and perceptions around nutritional health and wellbeing among UK food manufacturing shift work employees; (ii) externally validate these findings with industry stakeholders; and (iii) identify priorities to take forward for research, policy and practice. The Theoretical Domains Framework (TDF) was selected to guide data collection and analysis as it provides a comprehensive, theoretically grounded approach to identifying behavioural determinants [7]. Its established use in qualitative dietary research with shift workers [8] and its direct mapping onto the COM‐B model [9]. While behaviour change frameworks have been applied in workplace health research more broadly, their systematic application in food manufacturing shift workers represents a novel contribution to this field.

## 2 Methods

Semi‐structured qualitative interviews were conducted with manufacturing staff employed at Marlow Foods Limited, the business partner on this BBSRC OIRC‐funded project. Marlow Foods Limited is a UK‐based manufacturer of meat‐alternative products. The company operates three food production sites in England. Semi‐structured interviews were selected in preference to focus groups, as they allow greater flexibility to accommodate participants' shift‐working patterns. The study was conducted through an interpretivism research paradigm. This approach assumes that people's knowledge of reality is formed through social interaction and focuses on the interpretation of behaviours and perceptions to gain an understanding of individual motivations [10]. Study reporting is in line with the consolidated criteria for reporting qualitative research (COREQ) [11] (Supporting Information: S1).

### 2.1 Research Ethics Approval

Ethical approval was obtained from King's College London Research Ethics Subcommittee LRS/DP‐24/25‐47793 and registered at OSF https://doi.org/10.17605/OSF.IO/84F3M. Informed consent was obtained digitally. Informed consent was also verbally obtained from the participant at the commencement of each interview.

### 2.2 Participants and Recruitment

Eligible participants worked night shifts as part of a mixed shift schedule at Marlow Foods Limited. Inclusion criteria were having worked at Marlow Foods Limited in one of their three manufacturing sites for a minimum of 1 month and having access to a personal electronic device with internet connection to carry out the interview. Participants were recruited through advertisements via internal email and e‐posters displayed on notice boards in the worksite production and staff ‘mess’ areas. Potential participants were able to express interest in taking part by directly contacting the research team via a QR code linked to an online form or by telephone. A pragmatic decision to use convenience sampling was taken. Participants were offered £50 compensation for their time; payment was in online shopping vouchers. Recruitment and interviews took place between May and July 2025. A sample size of 15 participants was estimated to be required based on the principle of thematic data saturation [12], the point at which no new themes are derived from subsequent transcripts. Based on the three different geographic sites, a recruitment target of six to eight participants from each site was used in the study protocol.

### 2.3 Interview Materials

An interview topic guide was developed (G. R., R. G., E. P., W. H.). The topic guide (Supporting Information: S1) included initial rapport‐building questions about the employee's job role and working pattern. The main part of the interview was to explore factors influencing healthy nutrition choices when at work (e.g., the target behaviour) and to understand perceptions of worksite nutritional health and wellbeing provision. The guide was informed by a comprehensive scoping review and an industry practitioners survey [6]. The topic guide was circulated to the project steering group for comment and piloted prior to commencement of data collection with a Registered Nutritionist (G. R.) with experience in UK manufacturing employee nutritional health. BRH reviewed the study materials; their input was limited to refinement of wording to ensure relevance to the target participant group. B.R.H. did not influence the questions asked or take any role in data coding and analysis.

### 2.4 Data Collection Procedure

Prior to the interview, participants completed a brief online survey to record demographic and occupational data. Interviews were conducted via one‐to‐one video call using Microsoft Teams (version 25227.203), outside of their working hours and at a time and date in accordance with participant preference. Participants were advised that interviews would take a maximum of 1 h. All interviews were facilitated by a female Research Assistant (G. R.), a Registered Nutritionist with training in qualitative data collection methods. G. R. has an interest in workplace nutritional health and has previously worked in the delivery of corporate workplace nutrition programmes. She has been involved with several research projects investigating workplace nutritional interventions and health. No prior relationship existed between the interviewer and participants, and no follow‐up interviews were undertaken. No one else was present during the interviews besides the researcher and the participant. Interview data were recorded and transcribed verbatim by a third‐party professional transcription service and checked for accuracy (G. R.). All data were anonymised and securely stored on a digital platform for analysis. Transcripts were not returned to participants for comment. No field notes were made during or after the interviews. A data saturation monitoring approach was taken. No new points were made after the 8th interview, indicating thematic saturation and recruitment was stopped at 11 participants.

### 2.5 Data Analysis

Transcript data were analysed using NVivo 2025 (QSR International Pty Ltd) and Microsoft Excel. A combined deductive framework and inductive thematic analysis approach was followed: (i) familiarisation – transcripts were read and re‐read; (ii) a codebook was developed around the domains of the TDF [7] (this included definitions for each of the TDF domains and illustrative quotes to guide deductive coding); (iii) individual analysis of each transcript, with participant responses deductively coded to the TDF framework; (iv) inductive thematic analysis was then applied to responses within each domain, generating theme labels that captured the role of each domain in shaping the target behaviour; (v) the key domains influencing the target dietary behaviour were identified. Data were coded by one researcher (G. R.) and reviewed by another researcher (R. G.); discrepancies were resolved by consensus. Participants did not review or provide feedback on the findings.

The TDF domains were subsequently mapped to the COM‐B model (Capability, Opportunity, Motivation – Behaviour), which provides a higher‐order framework for understanding behaviour change [13]. Specifically: C – Capability includes domains such as Knowledge, Skills, Memory, Attention and Decision Processes, and Behavioural Regulation; O – Opportunity encompasses Environmental Context and Resources and Social Influences; and M – Motivation includes domains such as Beliefs about Capabilities, Beliefs about Consequences, Intentions, Goals, Reinforcement, Emotion, and Optimism.

### 2.6 Mapping to Intervention Strategies and the Social Ecological Model (SEM)

To generate suggestions for potential ways to support dietary behaviour change in this context, the themes generated from the qualitative analyses were then mapped to potential Behaviour Change Techniques (BCT), following published approaches [14]. Potential BCTs were discussed by the research team to generate recommendations and examples for how these could be operationalised and delivered in the context of food manufacturing shift workers. To determine likely stakeholder involvement for interventions, the findings from the TDF and COM‐B analysis were mapped onto the SEM [15]. The SEM conceptualises behaviour as influenced by multiple, interacting levels of influence: individual, interpersonal, institutional, community and policy levels, and can support stakeholder intervention identification.

Prioritisation of TDF domains to be taken forward for stakeholder mapping was determined by the frequency of participant reference and the salience of content within transcripts. Domains referenced by multiple participants were retained as key influences on the target dietary behaviour. Intervention strategies were then mapped to these prioritised domains through consensus discussion within the research team.

### 2.7 External Validation – Workshop

The results of the prior scoping project and semi‐structured interviews were presented by research team members (G. R., R. G., B. R. H.) at an in‐person stakeholder workshop. Stakeholders with direct or indirect influence on workplace nutritional health of UK food and beverage manufacturing staff were invited to take part in a workshop held at The British Dietetic Association Offices, Birmingham, UK. The workshop was attended by 11 participants. The SEM framework was used to present summary findings. This framework was used to guide group discussion and activities during the workshop. The research team recorded written notes throughout the day, with findings subsequently circulated to all attendees for approval [16].

### 2.8 Trustworthiness

We ensured trustworthiness using Lincoln and Guba's [17] criteria of credibility, dependability, confirmability, and transferability. Credibility was supported through repeated engagement with the data and team discussions, and discussion of our findings with stakeholders at the workshop. Dependability was strengthened by maintaining an audit trail detailing analytic decisions and coding development, enabling transparency and procedural consistency. Confirmability was addressed through reflexive notes and G. R. reflections to minimise researcher bias. Transferability was facilitated by providing descriptions of the study context, sampling approach, and participant characteristics to enable applicability to other settings.

## 3 Results

### 3.1 Participants

Eleven interviews (mean duration 37 min, range 27–53 min) were conducted. All participants worked full‐time (> 35 h per week) at the same worksite. No participant terminated the interview or withdrew their data. Most participants (*n* = 7, 64%) worked as production operatives or quality assurance technicians. Participants had worked atypical hours for an average of 14 years (range 2–35 years) and currently worked night shifts as part of a 2 nights, 2 days mixed shift pattern. Ten participants (91%) were male, and 10 participants (91%) were White. Summary of participant characteristics is shown in Table 1.

**Table 1:** Participant characteristics of those completing the semi‐structured interviews.

	*n*	%
Job role		
Quality assurance technician	4	36
Production operator	3	27
Other: Food worker clean‐down, Shift manager, Maintenance operator, Manufacturing technician	4	36
Full‐time worker	11	100
Male	10	91
Age range (years)		
25–34	3	27
35–44	2	18
45–54	3	27
55–64	3	27
Ethnicity		
White^a^	10	91
	Mean	Range
Total time working shifts, years	14	2–35
Time at Marlow Foods (years)	7	3 months to 27 years

^a^ One participant did not identify as White, to protect from identification their ethnicity is not reported.

### 3.2 Influences on Dietary Behaviour During Shift Work – Emergent Themes

**Figure 1:**
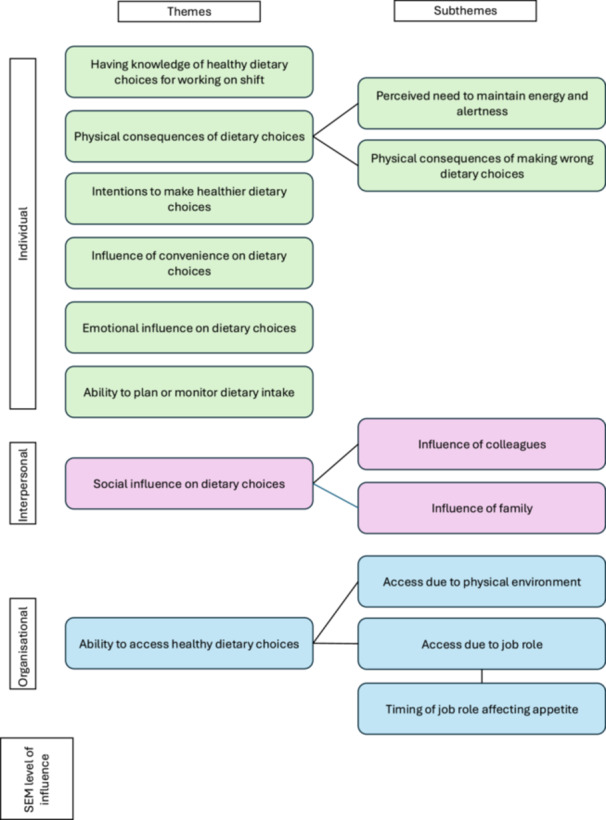
Influences on healthy dietary choices when at work; emergent themes and subthemes arranged by the Social Ecological Model level of influence.

Eight themes were identified as influencing healthy nutrition choices for participants when at work (Figure 1).

Themes were mapped onto 8 of the 14 TDF domains. The most referenced domains were: *Environmental Context and Resources*, *Beliefs about Consequences*, *Memory, attention and decision processes*, *Knowledge*, *Social influences*, and *Behavioural regulation*. Table S1 shows the full list of themes generated within each domain. A narrative summary of findings by the TDF domain is outlined below with representative quotations across a range of participants.

#### 3.2.1 Environmental Context and Resources

Within this domain, three subthemes were identified: Ability to access healthy dietary choices due to physical environment (this subtheme includes availability of food or supplies), ability to access healthy dietary choices due to job role (this includes working hours, breaks, job demands and regulations), and timing of job role affecting appetite.

##### 3.2.1.1 Ability to Access Healthy Dietary Choices due to Physical Environment

Influences identified within this subtheme were largely barriers. Many respondents reported not being able to access healthy food options in their workplace while on shift due to a lack of canteen facilities ‘There's not the facility, like there's not the space’. It was also mentioned that no packaged food purchasing options were available on‐site, particularly following the removal of vending machines.

Sometimes you wake up, you can't really go to the shop, you don't have time if you're, like, before the shift, so sometimes you might go without or order a takeaway.

[P08]

Most participants reported free fruit deliveries were provided by their employer – an enabler to support healthy food choices; however, most mentioned that the fruit runs out quickly, with there often being none left for night shift workers.

They provide fruit on a number of days. But like I say, when you're on nights, the fruit's gone.

[P12]

Several employees mentioned that healthy Quorn meals are provided monthly, which were generally well appreciated – this was seen as an enabler to healthy food intake.

They do a Tasty Tuesdays thing. So, they'll cook Quorn curry, something like that, so it's a little bit healthier than an actual curry.

[P10]

Several participants mentioned that they were not easily able to make drinks due to a lack of supplies, such as cups or spoons, in the staff (‘mess’) rooms – this was a barrier to healthy beverage intake.

The cutlery keeps going missing and we've only had like one teaspoon, so that's doing everyone's cup of tea.

[P12]

The lack of availability of unhealthy snacks or vending while working night shifts was commented on as a positive, enabling influence on healthy food choices by a minority of respondents.

In some ways that's probably slightly positive in terms of you don't snack because you've only got what you've got.

[P15]

##### 3.2.1.2 Ability to Access Healthy Dietary Choices due to Job Role

Influences within this subtheme were largely barriers. Over half the respondents reported time constraints, such as not being able to take regular breaks or taking short breaks, as a barrier to eating and drinking well on shift.

I try and eat healthy, but at times, half an hour…. you go straight back on plant and you're rushed about. I'll just have a drink because I can't be bothered eating because I haven't got time to settle or anything.

[P12]

One employee reported that the staffing and break situation has improved recently.

Several participants reported the barrier that they relied on food choices that are quick and easy to make, as breaks do not allow long enough to cook and eat a proper meal.

I normally take things that's quite easy to make quickly. So, I often take sandwiches because you don't really get long enough to cook a proper meal. So, it's generally sandwiches, or a tin of soup is probably the hottest meal I'll take, cooking‐wise, because it's quick.

[P07]

Several employees mentioned that chilled or Marlow Food Limited products were provided in the staff mess rooms free of charge; however, most participants said they did not find them appealing, having worked closely with the product in production.

Where if you like around that food 12 hours a day you don't really feel up to eating it.

[P08]

Restrictions regarding bringing certain types of foods containing allergens or receptacles into the workplace (such as nuts or glass containers) were reported by several respondents.

I can't take to work and that's my enjoyment. To me nuts and that are all healthy for you but I know people have allergens to stuff like that.

[P12]

##### 3.2.1.3 Timing of Job Role Affecting Appetite

Influences within this subtheme were largely barriers. The majority of participants reported having a lack of appetite during the night.

It's hard to be eating chicken and eggs at three o'clock in the morning’ and ‘the last thing you want to do is go and start cooking a meal and sit down and eat a big meal.

[P09]

Three participants noted that night work had a neutral influence on their dietary intake as it did not affect their appetite. *‘*Because I'm more of a night person, it doesn't really bother me’ *[P10]*.

#### 3.2.2 Beliefs About Consequences

Within this domain, two subthemes were identified: Perceived need to maintain energy and alertness, and the physical consequences of making the wrong dietary choices.

##### 3.2.2.1 Perceived Need to Maintain Energy and Alertness

Opinions on the use of sugary and caffeinated drinks were widely reported; this was a mixed influence. Responses were polarised, with some employees regularly relying on caffeinated drinks to maintain energy and others choosing to avoid energy drinks due to the health consequences.

I keep away from things like that. I won't go anywhere near these energy drinks and stuff.

[P14]

We all have an energy drink in the bag just in case, do you know what I mean? I wouldn't have that during the day.

[P09]

##### 3.2.2.2 Physical Consequences of Wrong Dietary Choices

Influences within this subtheme were mixed. Several participants reported avoiding certain foods that are ‘heavy’ or ‘greasy and cheesy’ as they are harder to digest, ‘because it sits on your stomach’ and ‘makes me feel constipated from eating something heavy during the night’. Several respondents mentioned that ‘heavy’ meals made them feel sleepy.

So, on a nightshift, you probably eat less, and you probably do eat lighter things. The last thing you want in nightshift is a heavy meal, because you just want to go to sleep after.

[P14]

The undesirable physical consequences of eating ‘unhealthy foods’ (crisps, chocolate bars, ‘carbohydrate heavy stuff’) were identified as an enabler to making overall healthier food choices.

#### 3.2.3 Memory, Attention and Decision Processes

One theme was identified in this domain: Influence of convenience on dietary choices. This was largely seen as a barrier. In this commonly reported theme, over half the respondents reported a reliance on ‘quick’ and ‘easy’ convenience foods such as microwaveable or heavily processed products due to time limitations of shift work.

I get in after a shift, I just want to shower and chill out, see the kids and stuff, so I don't really want to prepare food, so I'd rather than just pick up stuff on the way to work.

[P08]

#### 3.2.4 Knowledge

One theme was identified in this domain: Having knowledge about healthy dietary choices for working on shift. All the influences reported were found to be enablers. Approximately half the participants mentioned trying to prioritise healthy eating generally, and particularly tried to ensure sufficient protein intake to fuel themselves through a shift.

…a lot of protein just to keep me fuller and give me a bit more strength.

[P11]

One participant noted the importance of keeping sufficiently hydrated, and several mentioned they take supplements (e.g., Vitamin D and ‘Full of Greens’) to improve their nutrient intake.

I just try and drink plenty of water, cold water, or whatever you know, flavoured water, instead of drinking pop.

[P12]

Some participants reported receiving an online workplace nutrition training programme.

So, we do basic nutrition training. So, vitamins, minerals, teaches people about macros, like carbs and stuff like that and protein. I believe it was called Vitamin Q [training module].

[P09]

#### 3.2.5 Social Influences

Within this domain, two subthemes were identified: Influence of colleagues and influence of family. The influence of colleagues was generally found to be a barrier, and the influence of family was an enabler to healthy eating.

##### 3.2.5.1 Influence of Colleagues

Several respondents reported unhealthy influences of colleagues, such as ‘cake culture’, while one noted a lack of camaraderie in the mess room during the night.

If they want a cake, they'll bring it in. If they want a donut, they'll bring them in for everyone.

[P09]

##### 3.2.5.2 Influence of Family

The positive influence of a spouse or children was reported by over a third of respondents. It was noted that having family members at home to help with food preparation, or simply to be a role model, was helpful.

The wife will say “right, I'm going to put the slow cooker on and help yourself to take some of that with you to work”.

[P11]

and ‘We always try and have healthy meals, like I say I've got young kids and stuff like that, but we like to treat ourselves as well.

[P08]

#### 3.2.6 Behavioural Regulation

One theme was identified in this domain: Ability to plan or monitor dietary intake. Over half of respondents reported that they do try to plan or monitor their dietary intake, which was found to be a mixed influence.

Because there's no canteen, so I'll spend a bit of time at home thinking about what I want to take for my next four shifts. And I'll go to the supermarket, and I'll buy what I've planned on eating for my next four days.

[P14]

I find it easier, because I know roughly how many breaks I'll get, depending on if it's days, nights or weekends. I'll know what I can bring’, ‘I take my time to plan it, I make sure that I eat everything in that box because I know it's nutritional.

[P09]

One participant noted they found it hard to effectively plan when they are tired from working shifts.

If you're coming off nights and you're trying to get meals prepped you want to go to bed, so it's not that easy.

[P03]

#### 3.2.7 Emotion

One theme was identified within this domain: Emotional influences on eating behaviour. All influences within this domain were seen as barriers to supporting healthy nutrition choices on shift. Several respondents reported a negative impact on their dietary behaviour due to feeling bored, tired or depressed when working at night.

Sometimes if it's been a slow night as well, I tend to eat a lot more, because of a bit more boredom I guess.

[P10]

When you're on a break like you're bored so you seem to eat.

[P07]

But on a night, there is the biscuits and stuff like that, because you need the instant gratification on a night don't you. It is a bit depressing on a night.

[P09]

Sometimes I don't even eat when I come home from work because I'm that tired as such.

[P12]

#### 3.2.8 Intentions

One theme was identified within this domain: Intentions to make healthier choices. The influences in this domain were largely found to be enablers. One participant mentioned they had been advised at an occupational health assessment that they were overweight, which had incentivised them to prioritise healthy eating and exercise.

I was really, really overweight. So, I took it upon myself to get myself a gym membership, start eating healthy and I lost three stone in two and a half months.

[P10]

Another reported intending to drink more water following an operation, although despite their best intentions, was finding it hard to do so.

My wife keeps on saying “You must drink water. You must drink water.” I'll take maybe a litre of water and sometimes I'll come back and there's probably a litre of water still left.

[P15]

### 3.3 Workplace Nutritional Health and Wellbeing Provision

A number of additional participant views were noted. These included perceptions of their current workplace wellbeing provision, suggestions for future initiatives, the impact of shiftwork on health, social and family life, and employers' responsibility for health.

#### 3.3.1 Perceptions of Current Workplace Nutritional Health and Wellbeing Provision

All participants reported receiving an annual or biannual occupational health assessment, this was generally well received however several employees noted that little feedback is given, rather a simple pass or fail, highlighting a lack of tailored advice at this point. Some participants mentioned completing an online nutrition training module, but several noted that they aimed to complete it as quickly as possible.

they give the training out but yeah, how many people are just going through and ticking it, you know.

[P09]

One participant mentioned seeing a water intake chart in the toilets, others mentioned healthy eating posters in the factory or had been given an example healthy recipe card for using Quorn products. One participant noted a lack of social support, with little communication between staff members at break or mealtimes.

On nightshifts, especially, there could be seven or eight people sitting in there, and nobody is speaking to each other. Every single one of them are on the phone. There's absolutely no communication.

[P14]

Several employees reported workplace provision of mental health support, but noted it was difficult to attend due to their shift pattern.

We have workshops on mental health first aid and things, but again, with me working shifts, it's very difficult for me to attend. And when you're on your four days off, do you really want to go back into work for a couple of hours when it's like eleven, twelve miles to drive.

[P15]

Several barriers to engagement in workplace health programmes were reported. Most participants suggested their supervisor would not be supportive of allowing them time to access health initiatives.

Not in work time, no, definitely not. They do not let you – like you having time off.

[P07]

Several respondents noted that it would be difficult to engage shift workers due to the timing and pattern of their job roles.

But with there being five different shifts, it just can't benefit everybody. It would be very hard for people to get onto things like that.

[P13]

Several employees mentioned that agency staff and production colleagues are hard to engage in workplace wellbeing programmes as they do not regularly use email within their job role. One respondent mentioned they would not be interested in participating in a workplace health initiative as they felt it would not be useful for them.

I don't pay much attention to this because I've got my own pattern, and I stick to this.

[P13]

#### 3.3.2 Employee Suggestions Regarding Future Workplace Nutritional Health and Wellbeing Provision

Many participants voiced their views and suggestions on what could be done to improve nutritional health at their workplace. These were mapped to the BCT taxonomy [12]. The most associated BCT categories were *shaping knowledge* (4.1), *antecedents* (12.1) and *social support* (3.1).

##### 3.3.2.1 Shaping Knowledge

Employee requests included greater access to dietary education and information, including in‐person nutrition sessions, recipe cards, and improved signage.

More advice, more people to speak to or who to ring or, like I say, more details, like for example healthy eating day, this is what we're going to put on, or this is who to see or who you can contact, like a nutritionist for better advice of actually eating healthier’ [P03] and ‘We do have noticeboards in the mess room, but they don't really get utilised. So, the things that do go up there, they do get looked at.

[P10]

##### 3.3.2.2 Antecedents

Improvements to the physical landscape, such as hot canteen food and increased fruit deliveries, were frequently mentioned.

It would be nice if the company did have a canteen. It would be nice, for hot meals. Previous places I've worked, we've had that. And we actually had meals through the night as well, ran through the night, so you could have a hot meal through the night.

[P14]

##### 3.3.2.3 Social Support

Several respondents suggested employee feedback sessions or consultation forums would be helpful.

Like if they came back with a bit of feedback and say “Right, we're going to do some changes, what would you think?”.

[P12]

…getting involved with all the teams and ask their opinion what they want to discuss and then they have a general like forum once a month to put their opinions across.

[P03]

Provision of team building activities to increase physical activity, such as walking, football or cycling and access to mental health support were frequently mentioned.

#### 3.3.3 Shiftwork Impact on Physical and Mental Health, Social and Family Life

The negative impact of working night shifts on physical and mental health and social and family life was reported by most participants. Physical health concerns are predominantly related to tiredness, struggling with sleep, difficulty in timing meals and taking medications. Mental health comments mainly focussed on stress and anxiety and subsequent problems with family life.

So obviously I can't take my pain meds on a night because it makes me sleepy so obviously, I can't run a machine, so I have to go on without any pain meds.

[P07]

Mentally and physically. Like I say, sometimes on my days off, all I do is sleep. But I try not to because I've got kids to look after.

[P12]

#### 3.3.4 Employer/Employee Responsibility for Health

Several participants voiced opinions on where the responsibility should sit for employee health. Generally, comments reflected the opinion that employees are ultimately responsible for their own health, but that their employer could do more to help motivate them.

No, I think it's my responsibility. No. I think it's mine. I think if you want to look after yourself you will. If you want to eat unhealthily you will.

[P10]

They should have a duty of care as well, the company, shouldn't they, to say right, you're not as fit as you should be or eating as healthy as you should be, try and follow these guidelines.

[P11]

### 3.4 Incidental Findings

Several participants brought up the topic of weight loss medications. Concerns were noted that colleagues taking GLP‐1 receptor agonists were not eating appropriately for the demands of physical work.

### 3.5 Stakeholder Workshop Validation and Mapping of Findings to Potential Intervention Strategies

The views and experiences of the industry stakeholders aligned closely with the themes from the semi‐structured interviews presented. Through mapping the influences on the target behaviour (making healthy nutrition choices when at work) and considering the wider context of perceptions around worksite nutritional health and wellbeing provision, a range of potential interventions were identified (Table 2). Based on the interview findings and confirmed by the stakeholder group, the primary barrier to address is physical access to healthy food at work. It is likely that organisational policy strategies would be needed that target accessibility and availability of healthier food choices during shifts (e.g., healthy choice vending machines, 24‐h canteens, provision of suitably timed breaks with adequate facilities to enable healthy dietary choices).

**Table 2:** Reported influences on dietary behaviour and proposed intervention strategies.

Reported influences on dietary behaviour	Corresponding TDF domain(s)	SEM level of influence	Proposed intervention strategy	Example operationalisation	Key considerations for success in this context
Having knowledge of healthy food and beverage choices when working on a shift	Knowledge	Individual	Education and planning resources	Create a set of resources to support workers in how to adjust their eating patterns according to shift schedules. Resources could include blank meal planning templates, recipes and suggestions for healthy swaps. To enhance credibility and engagement, resources such as preparing food in advance to bring to work could be accompanied by advice or demonstration from a qualified nutrition professional. Materials could be delivered in paper form, that is, a handbook, mess room posters, tabletop information or digitally, that is, an app or electronic signage.	Dietary information provided should be budget‐friendly, nutritionally and culturally appropriate Simple, clear messaging should suggest quick, transportable, easily prepared meal/snack options
Influence of colleagues on dietary choices	Social influences	Interpersonal	Team challenges and support	Introduce a dietary behaviour champion (supervisor/colleague/peer) to encourage taking regular allotted breaks and healthier dietary behaviour during night shifts. Establish team challenges and competitions to encourage healthier dietary habits for shift workers. Small wellbeing incentives (water bottle? lunchbox/meal prep container) for achievement/engagement.	Supervisor and managerial level support for the programme is essential Financial support to allow small incentives to be awarded for changing behaviours or outcomes
Ability to access healthy dietary choices due to the physical environment	Environment and context	Organisational	Increasing availability of healthier dietary choices during the night shift	Increase the availability of healthier dietary options during night shifts. This could include a staff canteen serving hot meals open 24/7, healthier food and beverage options in vending machines, providing subsidies for healthy food and beverage options, restricting availability of unhealthier options, and providing subsidised or discounted ‘healthier’ snacks.	Availability of healthy food and beverage options for all staff, regardless of shift pattern Ensure quantities and timing of fruit deliveries are sufficient to enable night‐shift workers to access
Ability to access healthy dietary choices due to job role	Environment and context	Organisational	Increasing accessibility of healthier dietary choices during the night shift	Trial focus groups/roundtable sessions with workers, supervisors and managers to discuss how to support employees in accessing allocated breaks during night shifts. Encourage breaks to be taken in dedicated mess rooms with adequate facilities to allow for refuelling and rest.	Ensure the timing of sessions that allow workers on all shift patterns to attend. Consider the provision of remote access for employees to join sessions from home

## 4 Discussion

This study aimed to explore the factors influencing healthy dietary choices at work and the current provision of workplace nutritional health and wellbeing programmes within the UK food manufacturing sector, with a particular focus on how well these initiatives address the needs of underserved or under‐engaged employee groups, such as male shift workers. The findings highlight a number of important considerations for improving the relevance, accessibility, and impact of workplace nutrition strategies in the context of this occupational group. Collectively, the findings suggest that dietary behaviours at work are not driven solely by individual choice, but arise from the dynamic interaction between individual capability, opportunity and motivation within a highly constrained organisational context.

A consistent theme across participant responses was the significant influence of the workplace environment and context on dietary behaviours. This lack of (physical) opportunity for shift workers to access healthy dietary options was identified as a substantial barrier to improving the target behaviour. Within the COM‐B framework, opportunity refers to external environmental factors that enable or restrict behaviour [9], and in this study, opportunity‐related barriers frequently outweighed individual intentions to make healthier food choices.

Similar structural barriers have been reported in previous qualitative studies of shift‑working populations, with limited break duration, constrained access to food outlets and fatigue‑related time pressure shown to override individual intentions to eat healthily [18]. Research with night‑shift workers has also shown that inadequate workplace food provision and restricted access to food storage and preparation facilities significantly limit healthy dietary choices [19]. Shift workers, therefore, face notable structural barriers to healthy eating, including limited availability of hot meals, restricted canteen hours, and a lack of healthy vending options. This supports findings from previous studies where environmental barriers such as food access and affordability have been identified as significant across a range of shift‐working occupational groups [20, 21]. From a socio‐ecological perspective, these barriers operate primarily at the organisational level, reflecting how institutional policies and resource allocation shape the food environment accessible to employees. Inequity of provision for night workers was also noted; for example, the free fruit supplied by the organisation (which was viewed as supporting healthy food choices) was frequently unavailable by the start of the night shift. Key findings from this study were job role‐specific barriers faced by UK food manufacturing workers. The nature of the work context provides limited time periods to access appropriate nutrition on shift. These constraints illustrate how work design and production demands can materially limit employees' physical opportunity to eat, independent of dietary knowledge or motivation. Qualitative research in healthcare workers – with comparable safety‑critical, time‑pressured environments to manufacturing – has reported that job demands often take precedence over eating, with professional responsibilities and lack of ability to leave the ward cited as contributing to missed meals and reliance on convenience foods or caffeine [22]. Specifically, in food manufacturing, the short timeframe provided for taking breaks is impacted by the need to relocate to mess areas and remove protective clothing. Inclusive focus groups (across employees, supervisors and management) to allow an opportunity for consultation were suggested as a potential strategy for improving access to adequate opportunities for healthy refreshment.

Improving social opportunity, through on‐site social networks or team building initiatives, was frequently noted by participants. This could be an important consideration for future intervention developments. Food is a key part of human interaction, and in agreement with evidence from the European Sustainable Workforce Survey, it was found that employees were more likely to eat fruit and vegetables and engage in physical activity when their colleagues encourage a healthy lifestyle [23]. The present study observed that ‘treat’ foods bought in to share were perceived as having a detrimental influence on the healthiness of food choices. This finding highlights the dual role of workplace social norms, which may simultaneously support social cohesion while undermining healthy dietary behaviours. Conversely, participants also noted elements of isolation from colleagues when on shift, particularly during the night (e.g., interaction with phones rather than with their colleagues during breaks). Support from family or significant others was observed as crucial in order to cope with the diet and lifestyle disruptions of shift work.

In agreement with research conducted in UK healthcare workers [8], participants tended to demonstrate knowledge of what a healthy diet should look like. Participants demonstrated individual motivation to eat healthily during shift work, yet this was routinely undermined by structural deficits in physical opportunity, most notably the absence of canteen facilities, inadequate break provision, and inequitable access to healthy food during night shifts. These barriers operate primarily at the institutional level of the SEM, reinforcing that organisational‐level change is a prerequisite for individual behaviour change in this context. This finding reflects a key principle of behaviour change theory: improving capability without addressing opportunity constraints is unlikely to result in sustained behaviour change. For some, sound dietary knowledge did translate into healthy nutrition choices; with participants frequently noting the undesirable physical consequences of poor dietary choices as providing significant positive influence on their dietary behaviours. Some employees noted difficulty with effective meal planning, often impacted by fatigue from their shift pattern, leading to reliance on convenience products or caffeine to sustain them through their shift. Simple, nutritionally appropriate meal planning resources could be considered to support employees in a shift‐work context.

Fatigue actively drove poorer food choices, with participants reporting reliance on sugary snacks, caffeinated drinks and convenience foods as strategies to sustain energy and alertness through the shift. In addition, several participants highlighted that tiredness persisted beyond their shifts, impacting their motivation to make healthy food choices even on their days off. This concurs with a study in Australian night‐shift workers that identified prolonged fatigue limiting the ability and motivation of workers to engage in healthy eating [24] and qualitative studies of night shift workers, where fatigue has been shown to increase cravings for energy‐dense foods and to trigger increased caffeine consumption as a means of managing sleepiness [25]. These findings suggest that interventions targeting dietary behaviour in shift workers should address the emotional and psychological dimensions of night work alongside practical environmental barriers, for example, through the inclusion of sleep or mindfulness‐based components within broader workplace health programmes.

Despite most participants reporting receipt of annual occupational health assessments and online nutrition training, the perceived value of these was limited. This is consistent with the suggestion that tick‐box approaches to workplace health provision, without meaningful follow‐up or individualised guidance, are unlikely to provide employee wellbeing benefits [26]. Research identifies manager and supervisor support as a critical determinant of participation in workplace health promotion programmes, with low‐wage workers specifically found to be more reliant on supervisory permission to overcome coverage constraints that prevent participation ‐ a structural barrier that higher‐wage workers do not typically face [27]. This underscores the importance of embedding health promotion into working hours and organisational culture, rather than positioning it as an optional add‐on outside of shift time. Participants did express a desire to be actively consulted in the design and implementation of future interventions, with many offering specific suggestions. Aligning with broader evidence that participatory approaches foster greater ownership and uptake of health promotion activities [28].

### 4.1 Strengths and Limitations

This qualitative study enabled rich, contextual insights into the lived experiences of employees, particularly those working shifts and roles that are traditionally underrepresented in workplace health research. A strength of the study was the industry partner's input in ensuring the terminology of the interview questions was appropriate for the target population. Due to resources, the use of one independent coder and one verifier is noted as a limitation. The sample size was smaller than initially estimated and reflects the challenges of recruiting from this occupational group. While the study was advertised equally across the three worksites, all respondents came from the same, largest site. However, saturation of themes was observed after eight transcripts were coded. The study recruited a convenience sample, reflecting a limited demographic; however, the sample represented age and gender trends in the workforce on an amplified scale. For example, the factory floor workforce is 75% male, and the sample was 90% male. Age representation in the study is broadly the same as that of the factories. We do not have organisation‐specific data for workforce ethnicity split; however, industry‐sector data [29] suggest that approximately 40% of this occupational group were born outside the United Kingdom. The authors highlight as a limitation the low response rate among individuals from minority backgrounds, as the sample used in this study was restricted to specific UK food manufacturing plants. Therefore, the findings may have limited generalisability. Self‐selection bias needs to be considered – with employees who are more interested in health or more confident in using technology (interviews conducted online) opting to take part. The interviews were conducted only with staff employed at Marlow Foods Limited; perceptions around nutritional health and wellbeing provision in the workplace will be representative of that organisation. However, the external validation through a wider industry stakeholder group strengthens the confidence in the applicability of the derived themes to this target population group.

### 4.2 Implications for Practice and Research

A set of suggested interventions has been derived from the results of this project. Supporting the evidence for implementation of complex interventions to address health behaviours [30], a combination of organisational, social and individual strategies is likely required. The next stage would be co‐development of potential interventions with all stakeholders. To enable organisational support, there needs to be evidence that interventions to improve the nutritional health and wellbeing of UK manufacturing staff are of company benefit – for example, increasing staff productivity, recruitment and retention. This study aligns with the priorities outlined in the UK Government's Food Strategy for England [31], which emphasises the importance of improving dietary health across the population, particularly among underserved demographic groups. The strategy identified the need to reduce diet‐related inequalities and highlights workplaces as key settings for health promotion. The UK food manufacturing sector is essential not only for sustaining the national food supply but also represents a significant portion of the economy.

## 5 Conclusion

Multiple domains influence healthy nutrition behaviours for shift workers. This study has found that, in addition to previously reported barriers of physical access to healthy nutrition at night, UK food manufacturing staff face job role‐specific challenges, including adequate breaks, health and safety regulations and exposures to food production. Organisational policy and leadership will be essential to be able to develop effective intervention strategies. This project supports the development of a co‐designed, nutritional health and wellbeing programme tailored for UK food manufacturing staff.

## Author Contributions

R.G., W.L.H., B.R.H. and E.P. derived the research question and overall study design. GR, BRH and RG developed the topic guide. G.R. conducted the semi‐structured interviews. G.R. analysed the data. R.G. and G.R. drafted the initial manuscript. All authors critically reviewed the manuscript and contributed to the interpretation of the results. All authors reviewed the final version of the manuscript and approved it for publication.

## Conflicts of Interest

RG hold the following un paid roles: Research Lead, British Dietetic Association Work Ready Steering Group and Research Lead HercuWise Ltd. BR‐H is a current employee of Marlow Foods Limited and has previously worked for United Biscuits. LD and HT were employed at Marlow Foods Limited at the time of the research design. WH is a Consultancy for Zoe Limited.

## Supporting information


Supporting File

## Data Availability

The data that support the findings of this study are available on request from the corresponding author. The data are not publicly available due to privacy or ethical restrictions.
