# Supplementary material for: Supporting Nutritional Health of the UK Food Manufacturing Workforce: A Qualitative Study Exploring Insights From Employees
Source: J Hum Nutr Diet. 2026 Jul 9;39(4):e70310. doi: 10.1111/jhn.70310 (PMC13351476; doi:10.1111/jhn.70310)
Supplement: Supplementary file 1 — Supporting File [file JHN-39-0-s001.docx]

**Supporting nutritional health of the UK food manufacturing workforce: a qualitative study exploring insights from employees**

**Supplementary material**

**Topic guide**

The interviews are semi-structured, therefore precise wording, sequencing of questions, and use of follow-on prompts may vary from interview to interview to allow tailoring of questions to participant responses and maximise conversational flow.

**Pre-interview Introduction:**

*Thank you so much for agreeing to take part in this study and to speak to me today. Just to be clear from the start, your interview is totally confidential. XXXXXXXX are not aware who is taking part and will not have access to the information you provide.*

*Before we get started, I just have a couple of things I need to read out and double check from a research ethics point of view. So, first, can I please check that you are happy for me to audio-record this discussion so that I can focus on talking to you rather than taking lots of notes? Your audio transcript will be completely anonymised so that it isn’t possible to identify you or anyone else you mention.*

***Consent***

*Great. Can I also please confirm that you have read the study information sheet and know what these interviews are about? Do you have any questions?*

*Can I please confirm that you are giving your consent to be a part of it?*

***Preamble***

*Just to summarise, the purpose of the interview is to get a better understanding of your experience of working shifts and how this impacts your food choices, health and wellbeing, and how you view nutrition, health and wellbeing support at your workplace.*

*There are no right or wrong answers to the questions. I just want to hear your honest thoughts, opinions and experiences.*

*The interview is expected to last a maximum of one hour. This does depend on your responses but are you available for this length of time now?*

*As a reminder, this interview is meant to be confidential, so please refrain from using names where possible. However, following the interview, we will double check transcripts of our discussion and remove any identifying information so that everything is anonymous. You are also free to skip over any questions you don’t wish to answer, or to stop the interview at any time point.*

1. **General Occupation / Working pattern questions**
2. **Let’s start with some general questions about you and your work**

- What is your current job/role?
- How many hours do you work per week?
- How often do you have to work night shifts in this role at XXXXXX specifically? And how long (have you done this) for?
- Is this consistent or does this change often?
- How long have you been working night shifts more generally?
- How long is a shift?
- Do you get breaks during your shift? (How often/How long)?

1. **Work and health questions**

**Moving on to some questions about working and your wellbeing**

1. **How do you think your job role impacts your health and wellbeing?**

If no, prompt – do you think it might in the future? Or why do you think it has no impact?

If yes, prompt

- (If been doing shift work for long time) - do you find shift working has become more impactful on your health and wellbeing the longer you have been doing it?
- What aspects of the job role – activity / labour – nature of the work; environment (e.g., temp, production line); time of shifts, duration of shifts, demands of production

1. **How does your job role impact your food choices?**

- Prompt around times and types of meals, types of foods
- “"Do you think your eating habits affect how you feel at work? What about outside work? Does this bother you?"
- Possibly “How do you usually decide what to eat at work or after a shift?”
- What about healthy eating in life more generally?
- Prompt – how do your colleagues impact food choices
  - Probe – social aspects to eating on shift
- Prompt: What about fluid intake, do you feel your role impacts how much you drink on shift?
  - (probe) In what way?

1. **Compared to other things you must do during a shift, where does eating healthily** **rank in terms of priority?**

Prompt - Is it a concern? What is of higher/lower priority?

(probe) Why?

1. **How do the types of things you typically eat and drink on a shift make you feel?**

- Prompts: Do you feel the types of things you eat and drink are good for your health and well-being? Energy levels/ alertness?
- What are the advantages and disadvantages to the types of things you currently eat/drink on night shifts?
- What would you like to change?
- Are there any foods or drinks you avoid on a night shift (and why)?

1. **Do you think you know how to look after your health when you work?**

If no – what kind of advice and information would help? What format? (Take home messages/messages in the staff kitchen)

if yes, what advice have you been given and where was it from?

1. **Nutrition, health and wellbeing support at work questions**
2. **What nutrition, health, wellbeing support or programs are provided at your workplace that help (you) staff make healthier lifestyle or food choices?** Prompt – posters, campaigns, cafeteria advertising, group sessions, free fruit deliveries, education sessions
   1. **If available, do you take personally take part? / get involved**

If no – what would encourage you to take part? Prompt - incentives, time in working pattern to allow this, supervisor support or influence of colleagues etc.

If yes - what do you find helpful/unhelpful?

- 1. (Capability) **Do you think your workplace gives you the skills and knowledge to help you make healthier lifestyle or food choices?** Explore details
  2. (Opportunity) **Does your supervisor/colleagues allow you sufficient time to take part in the any health and wellbeing activities?** Explore details
  3. (Motivation) **Do you think your workplace motivates you to make healthier lifestyle or food choices?** Explore details

1. **Is there any other nutrition, health and wellbeing support that you would you find helpful at work?** Prompt –increased healthy vending options/more fridge or heating space/cooking demonstrations/staff challenges/health screening/awareness sessions?
2. **What is your opinion on your employer supporting you to make healthier lifestyle or food choices whilst you are at work?** Prompt – responsibility of employee/employer. Privacy

*Thank you very much for your responses so far. That concludes the questions I wanted to ask of you. Is there anything else you would like to add, or that you don’t feel has been covered by the questions I’ve asked so far?*

**Table S1 example code book by TDF domain**

| **SEM** | **COM-B** | **TDF** | **Theme** | **Subtheme** | **Barrier / enabler** | **Example quotes** |
| --- | --- | --- | --- | --- | --- | --- |
| Individual | Capability - psychological | Knowledge | **Having knowledge of healthy dietary choices for working on shift** | none | enabler | ‘I try and get enough protein in to help me do my job because I usually do like manual lifting or manual handling, the job that I do in my zone we have to be physically demanding. Pulling and pulling and stretching and doing, lifting different things to do our jobs, so we have to be, we have to prioritise what I take, so like chicken, make sure we've got protein in to make sure we can last the shift.’ |
| Individual | Motivation - reflective | Beliefs about Consequences | **Physical consequences of dietary choices** |  |  |  |
|  |  |  |  | Perceived need to maintain energy and alertness | mixed | ‘We all have an energy drink in the bag just in case, do you know what I mean? I wouldn't have that during the day.’ |
|  |  |  |  | Physical consequences of making wrong dietary choices | mixed | ‘On a night shift, I do try to make it light, like not so much a meal because, sorry but sometimes it makes me feel constipated from eating something heavy during the night’ |
| Individual | Motivation - reflective | Intentions | **Intentions to make healthier dietary choices** | none | Largely enabler | ‘So the way it was last year when I was eating healthy, I would like to get back to that again because I've kind of set myself another goal to lose another stone and a half this year, and it's not going very well.’ |
| Individual | Capability - psychological | Memory, attention and decision processes | **Influence of convenience on dietary choices** | none | Largely barrier | ‘I think if I wanted to, if I could be bothered, type of thing, I would do it, yeah, it wouldn’t be that hard to do because we have like air fryers and other things to cook food on and stuff like that if we wanted to, but like I say, I think it’s just more, I get in after a shift, I just want to shower and chill out, see the kids and stuff, so I don’t really want to prepare food, so I'd rather than just pick up stuff on the way to work.’ |
| Organisational | Opportunity - physical | Environmental context and resources | **Ability to access healthy dietary choices** |  |  |  |
|  |  |  |  | Due to physical environment (availability / supplies), | Largely barrier | ‘Sometimes you wake up, you can't really go to the shop, you don’t have time if you're, like, before the shift, so sometimes you might go without or order a takeaway.’ |
|  |  |  |  | Due to job role (breaks / work hours / demands of job / regulations) | Largely barrier | ‘So them things I can’t take to work and that’s my enjoyment. To me nuts and that are all healthy for you, but I know people have allergens to stuff like that you know.’ |
|  |  |  |  | Timing of job role affecting appetite | Largely barrier | ‘And then my appetite during the night shift is usually just something quick because I don’t have that, like I say, appetite to eat something properly. So, it definitely impacts whilst I'm at work. I don’t have – I’ll rarely have anything nutritional.’ |
| Interpersonal | Opportunity - social | Social influences | **Social influence on dietary choices** |  |  |  |
|  |  |  |  | influence of colleagues | barrier | ‘They like to bring cakes in, and if they want a cake, they'll bring it in. If they want a donut, they'll bring them in for everyone. And then because we have the kitchen, they constantly come down with, so I was constantly eating.’ |
|  |  |  |  | influence of family | enabler | ‘The wife will say ‘right, I'm going to put the slow cooker on and help yourself to take some of that with you to work’ and there’ll be a stew or there’ll be a curry or a chilli.’ |
| Individual | Motivation - automatic | Emotion | **Emotional influence on dietary choices** | none | barrier | ‘Yeah. But on a night, there is the biscuits and stuff like that, because you need the instant gratification on a night don't you. It is a bit depressing on a night, you know what I mean, on a weekend.’ |
| Individual | Capability - psychological | Behavioural regulation | **Ability to plan or monitor**  **dietary intake** | none | mixed | ‘Obviously, I always take my own food to work, because there's no canteen. So, I'll spend a bit of time at home thinking about what I want to take for my next four shifts. And I'll go to the supermarket, and I'll buy what I've planned on eating for my next four days. Healthy food is always on my mind. I always buy fruit. I always try and eat bread, wholemeal bread and stuff like that. So, I do try and take the right things to work, because it's a long time, twelve hours as well. It's very tiring.’ |
